# To Go or Not to Go: Degrees of Dynamic Inhibitory Control Revealed by the Function of Grip Force and Early Electrophysiological Indices

**DOI:** 10.3389/fnhum.2021.614978

**Published:** 2021-01-28

**Authors:** Trung Van Nguyen, Che-Yi Hsu, Satish Jaiswal, Neil G. Muggleton, Wei-Kuang Liang, Chi-Hung Juan

**Affiliations:** ^1^Institute of Cognitive Neuroscience, National Central University, Jhongli City, Taiwan; ^2^Department of Pathology and Laboratory Medicine, School of Medicine and Public Health, University of Wisconsin-Madison, Madison, WI, United States; ^3^Cognitive Intelligence and Precision Healthcare Center, National Central University, Jhongli City, Taiwan; ^4^Institute of Cognitive Neuroscience, University College London, London, United Kingdom; ^5^Department of Psychology, Goldsmiths, University of London, London, United Kingdom; ^6^Department of Psychology, Kaohsiung Medical University, Kaohsiung, Taiwan

**Keywords:** inhibitory control, selective stop-signal task, force, partial response, ERM, LRP

## Abstract

A critical issue in executive control is how the nervous system exerts flexibility to inhibit a prepotent response and adapt to sudden changes in the environment. In this study, force measurement was used to capture “partial” unsuccessful trials that are highly relevant in extending the current understanding of motor inhibition processing. Moreover, a modified version of the stop-signal task was used to control and eliminate potential attentional capture effects from the motor inhibition index. The results illustrate that the non-canceled force and force rate increased as a function of stop-signal delay (SSD), offering new objective indices for gauging the dynamic inhibitory process. Motor response (time and force) was a function of delay in the presentation of novel/infrequent stimuli. A larger lateralized readiness potential (LRP) amplitude in go and novel stimuli indicated an influence of the novel stimuli on central motor processing. Moreover, an early N1 component reflects an index of motor inhibition in addition to the N2 component reported in previous studies. Source analysis revealed that the activation of N2 originated from inhibitory control associated areas: the right inferior frontal gyrus (rIFG), pre-motor cortex, and primary motor cortex. Regarding partial responses, LRP and error-related negativity (ERNs) were associated with error correction processes, whereas the N2 component may indicate the functional overlap between inhibition and error correction. In sum, the present study has developed reliable and objective indices of motor inhibition by introducing force, force-rate and electrophysiological measures, further elucidating our understandings of dynamic motor inhibition and error correction.

## Introduction

The ability to inhibit a pre-potent motor response to adapt to sudden changes in the environment is an important function of executive control. Deficits in motor inhibitory control have been associated with several clinical disorders such as attention deficit hypersensitivity (Armstrong and Munoz, 2003), Parkinson’s disease (Obeso et al., 2014; Manza et al., 2017), and Tourette’s syndrome (Li et al., 2006; Wylie et al., 2013). The performance of inhibitory control can be derived from the independent horse race model (Logan and Cowan, 1984; Verbruggen and Logan, 2009; Logan et al., 2014; Schall et al., 2017). According to the model, go and stop processes are independent of each other. Once the primary go response is initiated, it will enter a ballistic phase that is called the “point of no return.” Stop-signals that appear later will not have any effect on the primary task response. Therefore, the independent race model suggests that the race occurs in an all-or-none fashion and unsuccessfully inhibited responses are assumed to be the same as a go response. However, several studies (de Jong et al., 1990; McGarry and Franks, 1997) have indicated that motor inhibition may not necessarily occur in such an all-or-none manner and this position may have originated due to the mode of responses collected from several motor inhibition studies that primarily involved key presses (Joundi et al., 2012). These studies show evidence against a ballistic stage because the response execution could be interrupted or modified (de Jong et al., 1990; Scangos and Stuphorn, 2010; Schultze-Kraft et al., 2016). Furthermore, it has been suggested to view the inhibition process as a disruptive process rather than in an all-or-none fashion.

The processes of motor inhibition are often investigated experimentally using the stop-signal task (Logan and Cowan, 1984; Boucher et al., 2007; Verbruggen and Logan, 2008). This task includes “go” and “stop” conditions. In go trials, the participant is required to respond to an imperative stimulus as quickly as possible. In some trials, there is a signal (stop-signal) presented after the go signal, often with a variable time delay, to instruct the participants to withhold their responses. In most stop-signal studies, the response is collected in a dichotomic fashion, no response (successful trials) or full response (unsuccessful and go). It is apparent that any inhibitory response which could not complete its course, that is a partial response or late and fractional motor inhibition, could not be measured by a conventional key press. Consequently, it is critical to develop objective indices that can measure partial responses with force measurements or electromyography (EMG; de Jong et al., 1990; Ko et al., 2012). Thus, the role of disruptive processes in inhibition led us to propose that motor inhibition is graded in nature rather than all-or-none. The response forces have been objectively used to measure the graded nature of motor inhibition (Ko et al., 2012). The present study employed a grip force device (The Pinch/Grip Analyser, MIE Medical Research, UK) to more precisely measure the stopping process. This allows measurement of partial responses, as well as additional indices such as force and force rate, in addition to the reaction time and accuracy measures (Joundi et al., 2012). Moreover, the appearance of partial responses illustrates that participants may inhibit error response activations before proceeding to full responses. A smaller force in error responses than for correct responses suggests that participants may be inhibiting an error response as while is being executed (Carbonnell and Falkenstein, 2006). The first goal of the current study was to replicate the previous study by Ko et al. (2012) wherein the non-canceled force (i.e., the force of responses made on failed stop-signal trials) was significantly smaller than the force for correct go responses. It was also observed in the same study that some aspects of the response could still be affected by inhibition even when the stop-signal appears too late to prevent the response being made. The main purpose of using grip force in the current study was to develop new measurement indices of motor inhibition which could reveal graded information about motor processing and timing with force and force rate measurements. It was hypothesized that the peak force and peak force rate of unsuccessful stop trials (USST) would increase as a function of the stop-signal delay (SSD). When the delay between a go stimulus and stop-signal is short (e.g., 90 ms), participants could mostly stop their response. At a longer delay (e.g., 180 ms), presumably, the stop process starts too late to allow withholding of the response by participants.

On the other hand, the stop-signal appearing abruptly during a stop trial in the conventional stop-signal task itself may evoke both attentional capture (i.e., the onset of an extra signal compared to go trials) and response inhibition processes concurrently. To separate attentional capture from response inhibition, Sharp et al. (2010) used continued go trials (Cont_Go) in a modified version of the stop-signal task (selective stop-signal task). In these trials, an abrupt signal appears to instruct participants to continue their go response to mimic the attentional capture effect from the stop trials. In the same vein, the present study employed a selective stop-signal task, using Cont_Go trials as the baseline for stop trials to allow analyses of the mechanisms underlying inhibitory (stop trial) and imperative responses (Cont_Go and go trials) with force and EEG measures. Previous studies (Sharp et al., 2010; Lee et al., 2016) have reported that participants took a shorter time to respond to go trials than to Cont_Go trials. These observations suggested that preparation of the go response might not have reached the threshold for initiating the go response before the appearance of the Cont_Go signals. The Cont_Go signal may correct the ongoing response trajectory or re-initiate the go response, either of which may result in a longer response time. In light of this, we predicted that each “continue” signal delay (CSD, the delay between a go and Cont_Go stimulus) would affect the outcomes of motor behaviors including response times, force, and force rate.

Additionally, many studies have investigated the electrophysiological basis of motor inhibition involved in the stop-signal task by recording the lateralized readiness potential (LRP) and event-related potential (ERP) components which can provide better temporal measures of the underlying neural correlates of motor inhibition. For example, de Jong et al. (1990) observed that the amplitude of the LRP on successful stop trials (SST) was larger than that of USST, suggesting LRP might reflect response preparation and inhibition processes in the motor cortex. In general, the LRP is attributed to central preparation for executing hand responses in trials. Similarly, several reports have proposed that N2 and P3 ERP component amplitudes can reliably reflect inhibitory processes. A larger negative deflection of the N2 component has been observed in USST trials than SST trials after stop-signal onset (de Jong et al., 1990; Kok et al., 2004; Ramautar et al., 2004, 2006). Additionally, a higher P3 amplitude in SST than USST trials has been shown (Dimoska et al., 2006; Ramautar et al., 2006). However, the results from some studies show that the processes indexed by the N2 and P3 components in inhibition may not be specific. For instance, the N2 component has also been reported to reflect conflict detection (Nieuwenhuis et al., 2003; Donkers and van Boxtel, 2004; Yeung et al., 2004; Enriquez-Geppert et al., 2010). The N2 has also been compared with the error-related negativity (ERN), which is evoked in error trials (Donkers and van Boxtel, 2004). It is assumed that the decision to inhibit a response would occur within the latency of the inhibitory response estimated with the stop-signal reaction time (SSRT), which normally ranges from 200 to 250 ms. Kok et al. (2004) reported that P3 latency usually outlasts the SSRT, suggesting that P3 cannot entirely account for the inhibitory process since it may appear too late to be involved in inhibition. Similarly, in a go/no-go task, Roche and colleagues observed that the N2 latency was larger than the mean RT of an error response (Roche et al., 2005). Therefore, these findings imply that both the N2 and P3 components are temporally late for indexing the inhibitory process in the tasks. In contrast, Filipovic and colleagues have demonstrated that the amplitude of the N1 component preceding EMG activity associated with response in no-go trials was larger than that in go trials (Filipović et al., 2000). It has also been observed that there is a larger N1 component for SST trials than for USST trials (Bekker et al., 2005). Thus, it is plausible that inhibition-related processes can be better indicated by an early component such as N1. On the other hand, the inhibitory processes are associated with several cortical and subcortical structures, including the right inferior frontal gyrus (rIFG), the pre-supplementary motor area (pre-SMA), dorsal anterior cingulate cortex (dACC; Rushworth et al., 2002; Aron et al., 2004; Aron and Poldrack, 2006; Xue et al., 2008; Chen et al., 2009; Duann et al., 2009; Hsu et al., 2011; Juan and Muggleton, 2012; Neubert et al., 2013; Yu et al., 2015). In the current study, we also used source localization of ERP to identify the brain regions associated with inhibition or attentional capture in those areas.

As above mentioned, the temporal precision of the ERP components is critical for gauging the neural correlates of the processes involved in the stop-signal task. ERP analysis in the conventional stop-signal task has utilized a rather arbitrary categorization of “fast go trials”/“slow go trials” compared with successful stop trials and unsuccessful stop trials to derive the components (Kok et al., 2004; Lo et al., 2013). In contrast, in the current study, the Cont_Go trials can directly offer temporally precise timestamps for comparing ERP components between stop trials and Cont_Go trials. This means that ERP components from Cont_Go trials can serve as the baseline for stop trials for an objective comparison. It was predicted that the N1 component would reflect inhibitory control processes and the effects of Cont_Go signals on central motor processing would be revealed with the LRP. Furthermore, an effect of the Cont_Go signal on central motor processing was expected to result in differences in Cont_Go trials and in go trials for the LRP measure.

In sum, there are six primary aims of this study: (1) to reveal characteristics of full and partial USST; (2) to develop detailed objective indices of motor inhibition by introducing force and force rate measurement which may provide a gradient and finer estimate of inhibitory control processes; (3) to examine the influence of behavioral data such as RT, force or force rate on each CSD of Cont_Go; (4) to investigate the motor inhibition mechanism using a modified version of the stop-signal task which can allow separation of inhibition from attentional capture with the expectation that the early N1 component can serve as an index of inhibitory processes; (5) to reveal the brain regions related to inhibition process using source estimation of the ERPs; and (6) to investigate whether the Cont_Go signal affects central motor processing, indicated by the LRP index.

## Materials and Methods

### Participants

Twenty-three healthy right-handed undergraduate and graduate students (seven females) were recruited from National Central University, Taiwan. The ages of the participants ranged from 21 to 32 years (*M* = 23.7, *SD* = 3.4) and all had normal or corrected-to-normal visual acuity. The experiment was approved by the Institutional Review Board of the Chang Gung Memorial Hospital, Linkou, Taiwan. Written informed consent was obtained from all participants before the experiment. Data from three participants were excluded because of high non-canceled rates on the task (two participants had a non-canceled rate higher than 90%) and an incomplete number of trials (one participant felt uncomfortable with the EEG cap).

### Apparatus and Stimulus

#### Apparatus

The experiment took place in a sound-attenuated room. Participants were seated 60 cm in front of a 23-inch LCD monitor which was positioned at eye level and on which stimuli were presented with a screen vertical refresh rate of 120 Hz. The task was programmed in MATLAB (R2014a) using Psychtoolbox-3 (PTB-3; Brainard, 1997; Pelli, 1997). The Pinch/Grip Analyser (MIE Medical Research, UK) was used to measure force and force rate (Joundi et al., 2012), with one force pincher held in each hand. The participants responded by pinching the force griper with their thumb and index fingers either with the left hand or the right hand, depending on the direction of the arrow of the task (see below for task description). Two force gripers were connected to the Biopac MP36 (Biopac Systems) to convert the grip signal from analog to digital using a 1,000 Hz sampling rate. This device was connected to a personal computer running MATLAB (R2014a), which recorded raw data from the two force gripers then processed the signal online to allow immediate determination of the response characteristics such as accuracy and determining if they were fast or slow responses.

#### Selective Stop-Signal Task

The stop-signal task had three types of trials: go, stop, and Cont_Go. A go trial began with a 500 ms central fixation of a white cross and it was followed by a 200 ms blank screen. Next, the go signal was presented for 1,000 ms and was followed by a blank screen with an inter-trial interval (ITI) of 1,500 ms. All stimuli were presented in the center of the screen. The go stimulus was either three “greater than” white symbols (⋙), with a visual angle of approximately 1.5°, indicating the rightward direction, or three “less than” symbols (⋘) indicating the leftward direction. When the arrows pointed to the right side, the participants were required to respond on the force pincher in their right hand. When the arrows pointed to the left side, they were required to respond with the pincher in their left hand. Participants were asked to respond as quickly and strongly as possible without sacrificing accuracy. In some trials, a dot appeared above or below the go stimulus, indicating that the participants were required to withhold their responses to the peripheral target. In the Cont_Go trials, a different colored dot was used as a Cont_Go signal and this was also presented above or below the go signal. The participants were required not to alter their actions and respond to the go stimulus. The dots for indicating stop and Cont_Go trials were either red or green and were counterbalanced across participants. The probability of a stop-signal and a Cont_Go signal appearing on a trial was the same, with both having a 25% likelihood (such that trials were 50% go, 25% stop, and 25% Cont_Go). The three types were pseudo-randomized to avoid more than either two stop trials or two continue go trials appearing in a successive sequence. The leftward and rightward go stimuli were equally frequent.

Overall the testing was divided into three sessions. A simple go RT session took place where the baseline mean RTs for each participant were measured. This was followed by determining the critical SSD (i.e., delay time for acquiring ~50% correct and ~50% error in the stop trials) for each participant. Finally, the critical SSD was applied to the formal task so that individual differences in critical SSD would not affect task performance. A detailed description of each session is as follows.

##### Session 1: Baseline Go RT Session

To prevent strategic slowing in the stop trials, we obtained each participant’s distribution of go RTs. Every participant started with a session of choice RT task (30 trials, go trials only). In this session, the task began with a fixation point. After that, there would be arrows pointing to left or right. The participants were instructed to respond as quickly and as hard as possible when the target appeared. The goal of this session was to acquire the baseline reaction time with grip force measures. It was also used to monitor the participant’s performance in the formal task sessions. In the following sessions, if the reaction time was two standard deviations longer than the mean baseline reaction time, indicating a strategic slowing, a visual feedback message of “Please respond faster” and a warning beep was be presented on the screen. This procedure has been previously demonstrated to limit the strategy of making intentionally slow responses to reduce non-canceled stop trials (Chen et al., 2008, 2009; Hsu et al., 2011). Having an objective time limit on making responses was used to evaluate baseline go RTs and also to constrain strategic slowing in an earlier study (Floden and Stuss, 2006).

##### Session 2: Critical SSD Session

This session aimed to estimate each participant’s critical SSD at which their non-canceled rate would be around 50%. The initial SSD was set at 170 ms and a tracking procedure was employed for acquiring the critical SSD. The program monitored the participant’s performance block by block, with each block comprising 32 trials (24 go trials). If the participant’s non-canceled rate was lower than 37.5%, the level of difficulty of the next block would be increased with a 40 ms increment of the SSD. If the non-canceled rate was higher than 62.5%, the difficulty level was reduced with a 40 ms decrement of the SSD. If the participant’s non-canceled rate was between these two levels then the SSD was not altered. The SSD had a minimum of 50 ms and a maximum of 290 ms. The critical SSD was determined when the non-canceled rate was between 37.5 and 62.5% for two consecutive blocks. This typically took less than 500 trials.

##### Session 3: Formal Experiment Session

**Figure 1:**
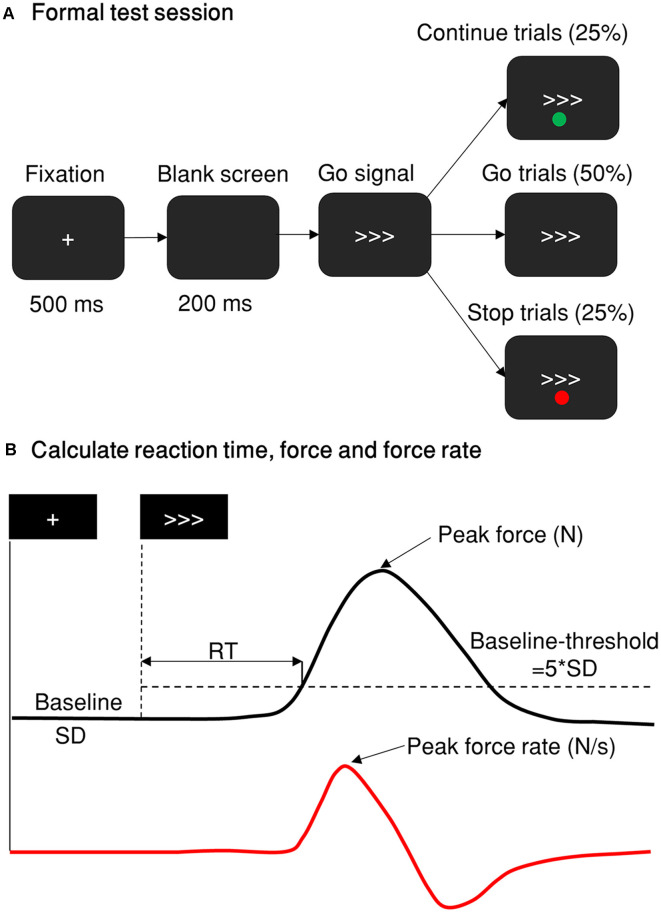
Stop-signal paradigm and behavior measurement. **(A)** The stop-signal paradigm comprises go, Cont_Go, and stop trials. The red and green dots indicating stop or Cont_Go trails were presented either above or below the go signal with their color/instruction counterbalanced across participants. The proportion of go, stop and Cont_Go trials were 50, 25, and 25%, respectively. **(B)** A typical trial response measured by a grip force device. The black line represents the force distribution of a single response of one trial. The peak force response was recorded as the maximum force within 1,000 ms of the go stimulus. If this was five standard deviations (SD, baseline-threshold = 5*SD) greater than the force in the baseline period (recorded from the fixation onset until the go signal onset), then it was defined and scored as a response. The reaction time was calculated as the time from go signal onset until the force level was more than five standard deviations above the baseline level. The red line denotes the force rate, or the gradient of the force level, obtained by differentiating the force distribution of the response. The peak force was measured in newtons (N) and the force rate was measured in newtons per second (N/s).

After obtaining each participant’s mean go RT and critical SSD, the formal stimulus-selective stop-signal task was carried out (Figure 1). Three fixed SSDs were used based on the individual critical SSDs obtained: (1) 40 ms shorter than critical SSD (SSD1); (2) critical SSD (SSD2); and (3) 40 ms longer than critical SSD (SSD3). For example, if a critical SSD was 90 ms (SSD2 = 90 ms), the other two SSDs used were 50 ms (SSD1 = 50 ms) and 130 ms (SSD3 = 130 ms). In the Cont_Go trials, the same delays were used and were referred at continue signal delays (CSDs). Therefore, the inhibition function and the continue-go function could be plotted to gauge participants’ capabilities to respond to the stimuli with various degrees of temporal urgency. At the beginning of each trial, the participants were required to keep their gaze on a fixation point for 500 ms. After that, a blank screen was presented (200 ms), followed by a go stimulus which consisted of three arrows pointing either to the left or to the right. Arrows pointing to the left indicated a response had to be made with their left hand, arrows pointing to the right indicated a response had to be made with their right hand. In some of the trials (stop condition), a red (or green, see above) dot which served as a stop-signal appeared at one of the three SSDs after the go stimulus to indicate that the response had to be withheld. In the Cont_Go condition, there was a green (or red) dot presented in the same manner as the stop-signal but indicating that participants had to continue to make a response. A total of 10 blocks of 64 trials each were presented, taking approximately 60 min to perform.

### Electroencephalography Recording

EEG was recorded from 36 Ag/AgCl electrodes (NeuroScan Synamp2) using standard positions according to the extended 10/20 system (Channels: FP1, FP2, F7, F3, Fz, F4, F8, FT7, FC3, FCz, FC4, FT8, T3, C3, Cz, C4, T4, TP7, CP3, CPZ, CP4, TP8, T5, P3, Pz, P4, T6, O1, Oz, O2, VEOU, VEOL, HEOL, HEOR, A1, A2). Electrodes were mounted on a plastic cap (Quick-Cap). The online reference was the average of electrodes at the left and right mastoids (A1 and A2), and the ground electrode was placed over Fz. The vertical and horizontal electrooculograms (EOG) were also recorded. Impedances of all electrodes were kept below 10 kΩ and data were recorded with Neuroscan 4.5 software, with a sampling rate of 1,000 Hz and without any band-pass filters for fully dimensional data analysis. All channels were re-referenced offline to the average of all channels.

### Data Analysis

#### Behavioral Analysis

For the go, unsuccessful stop, and Cont_Go trials, if the reaction time of the response was two standard deviations longer or shorter than the mean, the response value was considered as an outlier and was excluded from the analysis. Also, trials were rejected from behavioral analysis if the participant had responded either with the wrong hand or with both hands. The force and force race traces were aligned to their corresponding peak values to determine the peak force and the peak rate of force development respectively. SSRT was estimated using the distribution of go signal reaction times and non-canceled rate for a given SSD following the race model (Logan, 1994; Chen et al., 2008; Wang et al., 2013; Logan et al., 2014). Furthermore, in our experiments, USST trials were further categorized into partial and full USST trials according to the distribution of go peak force. First, the mean and standard deviation of the peak force of go were calculated. If the peak force of USST was smaller than (*M*– 3*SD) force of go, then it was defined as partial USST, otherwise, it was a full USST. The participant was excluded if the number of trials was less than six because these are fewer trials that have been shown to allow ERN quantification (six to eight trials, Olvet and Hajcak, 2009; Pontifex et al., 2010).

To compare the performances, a paired *t*-test was performed for the accuracy of Cont_Go trials and go trials. One-way ANOVA was conducted to compare the RT, force, and force rate of the go, USST, and Cont_Go trials. To account for novel indices of motor inhibition from force and force rate measurement, we separated the force and force rate of USST according to the three SSDs. The RT, force, and force rate of Cont_Go trials were also separated into each CSD to effectively assess how Cont_Go signals affect motor behavior. One-way ANOVA was performed to compare the reaction time, non-canceled rate, force, and force rate in Cont_Go and USST trials across the CSDs/SSDs. Because testing each effect (RT and non-canceled rate) repeatedly across three conditions could inflate the false positive rate, pairwise comparisons were performed with a Bonferroni correction for multiple comparisons. Results were considered significant at *p* < 0.05. The test was applied to force and force rate at each time point within the selected time windows ranging from −100 to 100 ms of the onset time of peak force and −50 to 50 ms of the peak force rate. Due to the high number of comparisons needed to generate the force and force rate for conditions, FDR correction was used for multiple comparison correction instead of Bonferroni correction to control the number of false-positive clusters.

#### Event-Related Mode Analysis

Several recent studies have applied event-related mode (ERM), an improved method for measuring ERP, to precisely explore task-related components (Williams et al., 2011; Al-Subari et al., 2015; Chang et al., 2016; Hsu et al., 2016; Chuang et al., 2019). ERM is based on empirical mode decomposition (EMD) or ensemble empirical mode decomposition (EEMD; Wu and Huang, 2009; Wu et al., 2009). EMD or EEMD is a fully adaptive and data-driven method to decompose non-stationary and nonlinear signals such as EEG into several intrinsic mode functions (IMFs) from high to low-frequency ranges with minimized distortion of waveforms (Huang et al., 2009). The procedure of EMD/EEMD has been taken as a bank of adaptively dyadic filters (Flandrin et al., 2004; Wang et al., 2010), resulting in narrow-banded IMFs. The procedure of ERM first takes a partial sum of the resulting IMFs for each trial. Then, similar to measuring ERPs, averaging the partial sum of IMFs across trials gives ERMs. The EMD/EEMD method results in ERMs with extraordinarily high signal-to-noise ratios and stronger effect sizes when a fewer number of trials were used in an ERP study (Williams et al., 2011) or mismatch negativity (Hsu et al., 2016). Besides, EEMD, which is a noise-assisted version of EMD, has also been shown to be more resistant to noise than EMD (Huang and Wu, 2008). Therefore, the current study employed the EEMD procedure for data analysis.

**Figure 2:**
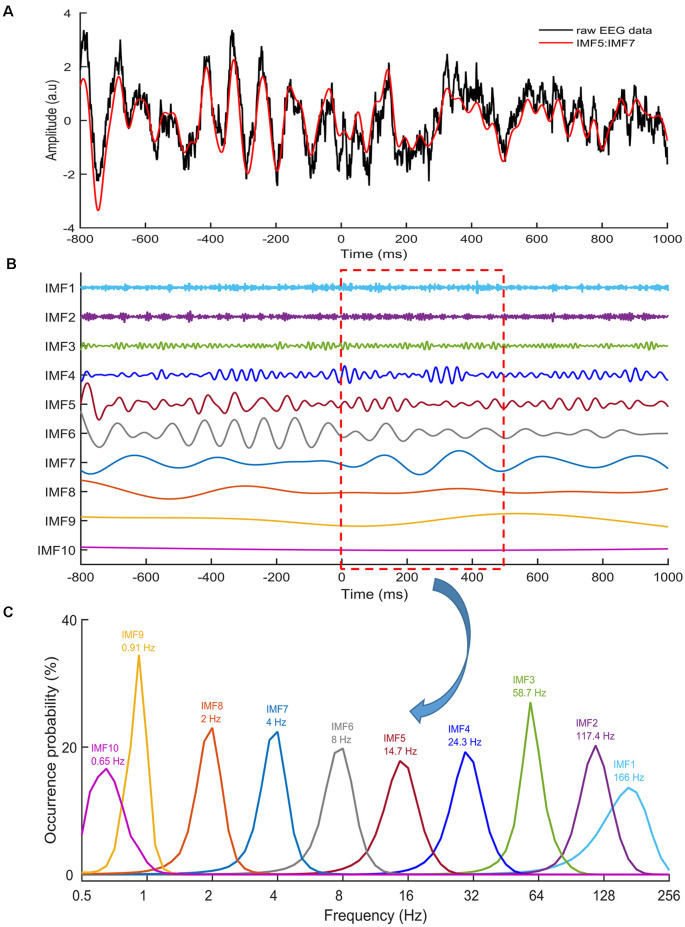
Intrinsic mode functions (IMFs). **(A)** An example of raw EEG data (black line) of a stop trial recorded from the Cz electrode. The red line denotes the sum of IMF 5,6 and 7. **(B)** Ten IMFs were obtained by using ensemble empirical mode decomposition (EEMD) for the EEG data, with Zero being the stop-signal onset. Different IMFs represented electrophysiological activities in different frequencies. **(C)** The distributions of instantaneous frequencies of each IMF for all stop trials from one participant from 0 to 500 ms. The results revealed that the IMF 8 showed a frequency range of 1–3 Hz, with a dominant frequency at 2 Hz. IMF 7 showed the frequency range of 2–6 Hz, with the dominant frequency at 4 Hz. IMF 6 showed the frequency range of 4–13 Hz, with the dominant frequency at 8 Hz. IMF 5 showed the frequency range of 8–24 Hz, with the dominant frequency at 14.7 Hz.

To estimate ERM in SST and USST conditions, continuous EEG data were segmented into epochs starting from 800 ms prior and 1,000 ms post stop/Cont_Go stimulus onset. An independent component algorithm was used to remove components associated with ocular artifacts and was followed by artifact rejection with a ±100 μV threshold for every channel (Spronk et al., 2008). Following artifact rejection, the EEG data of all trials in each channel were normalized by dividing by their standard deviation, resulting in a unit-free measure of amplitude. To obtain IMFs (see Figure 2), EEMD was applied for all epoched data with an ensemble size of 100 and a noise level of two (the ratio of the standard deviation of the added noise and the standard deviation of the original signal). In the event-mode analysis, the 5th, 6th, and 7th IMFs, which represented 4–14.7 Hz activity were selected to measure the N1 and N2 components (Chang et al., 2016). This avoided any possible overlapping with other ERM waves and were then summated for further analysis. Subsequent analyses were based on the IMFs between −300 and 500 ms of the stop-signal. The average ERM data was baseline corrected from −300 to 0 ms. Three conditions were tested in this experiment: Cont_Go trials, SST, and USST. The amplitude of each individual condition was averaged over the corresponding trials.

#### Error-Related Negativity (ERN) Analysis

The ERN is acknowledged as a frontal-central response and most ERN studies have focused upon the Cz channel (Davies et al., 2004; Wiersema et al., 2005). In this research, we reported here only data for Cz. To obtain the ERN in partial and full USST conditions, the 5th, 6th, and 7th IMFs were selected. Trials were averaged across participants using a window of 500 ms before response onset to 400 ms after response onset. The ERN for each participant was baseline corrected from −500 to −200 ms.

The ERN amplitude was obtained by the peak-to-peak difference between the amplitude of the positive peak that immediately preceded the ERN component and the negative peak, which accounts for the potential influence of the preceding positivity. The amplitude of the positive peak was calculated as the mean amplitude in a 40-ms time window centered at the latency of the positive peak for each condition. The negative peak latency of the ERN was defined as the timing of the negative peak during the time window from the onset of the responses to 200 ms. The amplitude of the negative peak was determined by the mean amplitude (±10 ms) around its latency.

#### Source Localization of ERM

Source analysis was applied to better understand the neural correlates of inhibition processes reflected by N1 and N2 components. A linear constrained minimum variance beamformer algorithm (Van Veen et al., 1997) was applied to sensor-level data to identify the sources of three conditions. A realistically shaped three-shell head model was derived from the Montreal Neurological Institute (MNI) template brain (Colin 27; Holmes et al., 1998). The lead fields for each grid point were measured using the boundary element method. The partial sum of IMF5 to IMF7 was projected into source space by multiplying it with the spatial accordant filter, resulting in source-level data at 1,963 virtual electrodes with a 10 mm resolution. ERM was calculated for the data from each of the virtual electrodes and parameters of ERM were the same at the sensor-level. All linear beamforming analyses were performed using the FieldTrip toolbox and custom MATLAB scripts.

To examine the time course and topography distribution, one-way ANOVA was performed on the data from the frontal and central electrodes (F3, Fz, F4, C3, Cz, and C4) for the time window of 0–500 ms after stop onset for each data point. FDR correction at a level of less than 0.05 was used for all time points and all six channels. To evaluate the ERM differences among conditions, the largest time window (for example N1 and N2) across six channels were selected. All time-points within the selected time windows were averaged across conditions, electrodes, and participants. A cluster-based non-parametric permutation (CBnPP) test was employed to test the differences in ERM between each of the paired conditions for sensor/source level in the N1 and N2 time window. In this study, if the distance between two sensors/sources was less than 70/20 mm, they were identified as neighbors. Five thousand permutations were performed for each test.

#### LRP Analysis

To assess the LRP, the average method proposed by Coles (1989), calculated by subtracting the ERP activity at C4 from C3 for right-hand responses; and C3 from C4 for left-hand responses was used. The difference in waveforms was then averaged. The 5th, 6th, 7th, 8th and 9th IMFs representing 0.9–14.7 Hz activity (see Figure 2) were selected and summated for LRP analysis. The LRP waveform for each trial (full USST, partial USST, go and Cont_Go) was locked to the response onset. There was no manual response in the SST trial. To compare the LRP waveforms with other conditions, the SST LRP waveform was temporally aligned at the time point of (SSRT + SSD2).

To account for the LRP deviation from the baseline and to test the condition-wise differences, a cluster-based non-parametric permutation test was employed with a sliding time window of 20 ms and step of 10 ms (each time window had an overlap of 10 ms with the preceding window). Condition-wise LRP waveform differences were analyzed in the 300 ms interval (150 ms before and after the onset).

## Results

### Behavioral Results

#### Partial and Full USST

**Figure 3:**
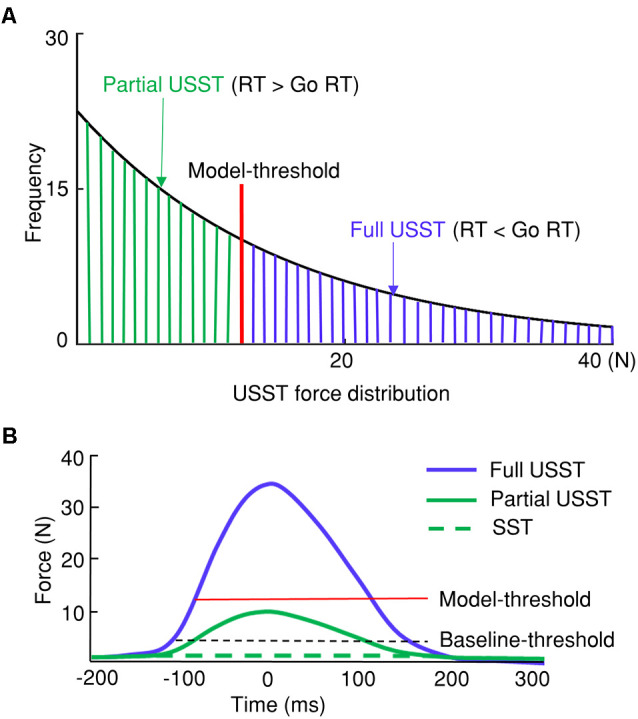
Categorization of stop trial responses. **(A)** Differentiation between partial and full unsuccessful stop trials (USST) with the USST force distribution. The model-threshold was (*M* − 3*SD) of go force was used to separate partial and full USST. **(B)** Thresholds determining successful stop trials (SST), partial, and full USST trials with the force measure.

In the current study, USST trials with their corresponding pinch force were categorized into full and partial USST trials. The current study proposes pinch force as a primary measure that can differentiate partial and full USST by (*M*– 3*SD) go force (model-threshold). The USST trials in which the peak force was smaller than the model-threshold were defined as partial USST and the trials otherwise were defined as full USST (Figure 3). Upon utilizing this gage, we found that the full USST RT was shorter than the go RT. In other words, our analysis meets the assumption of the horse race model [where the USST RT is faster than go RT (Bissett and Logan, 2014)]. Thus, the threshold to separate partial and full USST (model-threshold) was (*M*– 3*SD) of go force in the current study.

For partial USST condition, one participant was excluded since the number of trials was less than six. The RT of full USST (*M* = 335.0, SEM = 8.5) was faster than go RT (*M* = 350.5, SEM = 8.5), *t*_(19)_ = −2.179, *p* = 0.042, while the RT of partial response (*M* = 378.0, SEM = 11.8) was relatively the slowest in comparison with both go trials, *t*_(18)_ = 3.278, *p* = 0.004, and full USST trials, *t*_(18)_ = 6.859, *p* < 0.001. The RT of Cont_Go response (*M* = 408.4, SEM = 11.1) was significantly slower than RT of partial USST trials, *t*_(18)_ = 4.675, *p* < 0.001, and the RT of full USST trials, *t*_(19)_ = 12.095, *p* < 0.001. Moreover, the peak force (*M* = 38.0, SEM = 2.3) of full USST was larger than that of partial USST (*M* = 7.9, SEM = 1.4), *t*_(18)_ = 17.940, *p* < 0.001, and smaller than peak force of go (*M* = 45.8, SEM = 2.2), *t*_(19)_ = −10.237, *p* < 0.001. The peak force rate (*M* = 421.1, SEM = 23.1) of full USST was larger than partial USST (*M* = 137.2, SEM = 18.5), *t*_(18)_ = 15.310, *p* < 0.001, but smaller than the peak force rate of go (*M* = 459.9, SEM = 20.3), *t*_(19)_ = −5.324, *p* < 0.001 (Table 1).

**Table 1:** RTs, forces, and force rates for Go, Cont_Go, and non-canceled responses.

	Go	Cont_Go	Full USST	Partial USST	USST
Accuracy (%)	96.8 [0.7]	96.8 [0.6]	–	–	–
RT (ms)	350.5 [8.5]^b,c,d^	408.4 [11.1]^a,c,d^	335 [8.5]^a,b,d^	378.0 [11.8]^a,b,c^	352.6 [9.3]^b^
PF (N)	45.8 [2.2]^c,d^	45.0 [2.3]^c,d^	38.0 [2.3]^a,b,d^	7.9 [1.4]^a,b,c^	24.2 [2.1]^a,b^
PFR (N/s)	459.9 [20.3]^c,d^	456.0 [21.9]^c,d^	421.1 [23.5]^a,b,d^	137.2 [18.5]^a,b,c^	295.1 [22.9]^a,b^

*Note: RT, reaction time; PF, peak force; PFR, peak force rate; USST including the full and partial response of USST; “–”, not applicable; Standard errors of the mean are shown in square brackets; “a” denotes a statistically significant difference in comparison to the go condition; “b” denotes a statistically significant difference in comparison to the Cont_Go condition; “c” denotes a statistically significant difference in comparison to the full USST condition; “d” denotes a statistically significant difference in comparison to the partial USST condition*.

**Figure 4:**
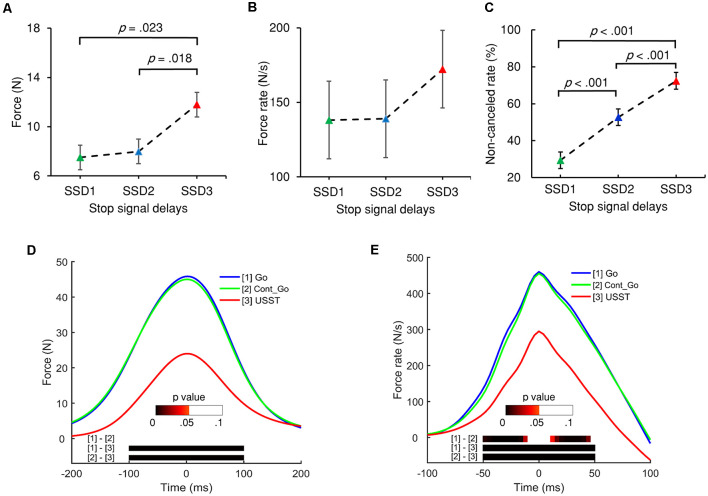
The peak force **(A)** and peak force rate **(B)** of the three stop-signal delays (SSDs) of partial USST. **(C)** Non-canceled rates for each SSD. Error bars indicate 95% confidence intervals for each SSD. All *p* values are Bonferroni corrected. **(D)** Grand averages of force and **(E)** force rate aligned to peak force/force rate for the three conditions: go trials (Go), Cont_Go trials (Cont_Go), and unsuccessful stop trials (USST). The color bars in graphs **(D,E)** indicate the level of *p*-value derived from the results of the *post-hoc* pairwise point-to-point comparisons (with FDR correction) across three conditions.

Each SSD of the partial USST was analyzed for its RT, peak force, and peak force rate. The partial USST occurred at 29.9, 40.5, and 29.5% on SSD1, SSD2, and SSD3, respectively. Data from eight participants were excluded because of the insufficient number of trials per SSD (less than six trials). There was a main effect for the partial USST peak force *F*_(1.235,13.581)_ = 10.096, *p* = 0.005 (partial *η*^2^ = 0.479). The SSD3 peak force was significantly larger than SSD1 (*M* = 7.5, SEM = 1.6), *p* = 0.023 and SSD2 peak force (*M* = 8.0, SEM = 1.7), *p* = 0.018. However, no significant difference was found between SSD2 and SSD1 peak force (Figure 4A). There was no significant difference in peak force rate values across the three SSDs (Figure 4B), *F*_(1.416,15.580)_ = 3.719, *p* = 0.060 (partial *η*^2^ = 0.253). It was also observed that there was no significant difference in the mean reaction times of each SSD of partial USST, *F*_(1.109,12.203)_ = 3.666, *p* = 0.076 (partial *η*^2^ = 0.25).

RT, force and force rate for full USST were also analyzed across SSDs. The full USST occurred at 11.4, 27.1 and 61.5% on SSD1, SSD2, and SSD3, respectively. Paired *t*-tests were applied only for SSD2 and SSD3 but not for SSD1 due to the inadequate number of SSD1 trials (13 participants). The peak force for SSD3 (*M* = 38.7, SEM = 2.7) was significantly larger than that of SSD2 (*M* = 36.0, SEM = 2.7), *t*_(13)_ = 3.739, *p* = 0.002. Also, there was no significant difference between SSD2 and SDD3 for reaction time, *t*_(13)_ = 1.779, *p* = 0.095 and force rate, *t*_(13)_ = 1.692, *p* = 0.115.

#### Replication of Previous SST Results

The accuracy of go RT and Cont_Go RT was not significantly different, *t*_(19)_ = 0.157, *p* = 0.877 and the error rate for both trial types was below 4%. The mean RT for USST (*M* = 352.6, SEM = 9.3) was not significantly different from that of go responses (*M* = 350.5, SEM = 8.5). The RT in the Cont_Go trials was longer than that of go and USST trials (*M* = 408.4, SEM = 11.1). In line with this finding, a repeated-measures ANOVA showed that the mean RTs were significantly different, *F*_(2,38)_ = 46.660, *p* < 0.001 (partial *η*^2^ = 0.711), with Bonferroni corrected *post-hoc* tests showing the mean RT of Cont_Go trials was significantly longer than the RT of both go trials (*p* < 0.001) and USST trials (*p* < 0.001). The RTs of go responses were faster than the RTs of partial USST (*p* = 0.004, paired *t*-test) and slower than the RTs of full USST (*p* = 0.042, paired *t*-test). In stop trials, non-canceled rates were 54.2%. A repeated-measures ANOVA showed that the non-canceled rate differed significantly between each SSDs, *F*_(1.727,32.813)_ = 91.326, *p* < 0.001 (partial *η*^2^ = 0.828) and increased with increasing SSD (see Figure 4C). The mean SSRT was 219.2 ms (SEM = 7 ms).

For the analysis of force, the continuous force value for the go, non-canceled, and Cont_Go responses were aligned to their respective peak force and analyzed with repeated measures ANOVA using point-by-point comparisons. The results indicated that the force and force rate value of go and Cont_Go responses were significantly greater than those of non-canceled responses. However, there was no significant difference for the force between go and Cont_Go in the 200 ms time window (Figure 4D). Furthermore, no significant difference in the force rate occurred at 15 ms around the peak value between go and Cont_Go (Figure 4E). To obtain detailed information about force and force rate measurements, the peak force and peak force rates of each response were analyzed. Significant differences for mean peak force, *F*_(1.030,19.570)_ = 88.432, *p* < 0.001 (partial *η*^2^ = 0.823) and peak force rate, *F*_(1,059,20.114)_ = 84.214, *p* < 0.001 (partial *η*^2^ = 0.816) for each condition were observed. On comparing the go and Cont_Go trials, neither peak force (*p* = 0.1) nor peak force rate (*p* = 0.234) showed any significant difference. On the contrary, the peak force and peak force rate of both go and Cont_Go responses were significantly larger than that those of non-canceled responses (all *p* < 0.001). Also, the peak force and peak force rate for both go and Cont_Go responses were significantly larger than that those for partial and full USST (all *p* < 0.001).

#### New Measures of Motor Inhibitory Control

**Figure 5:**
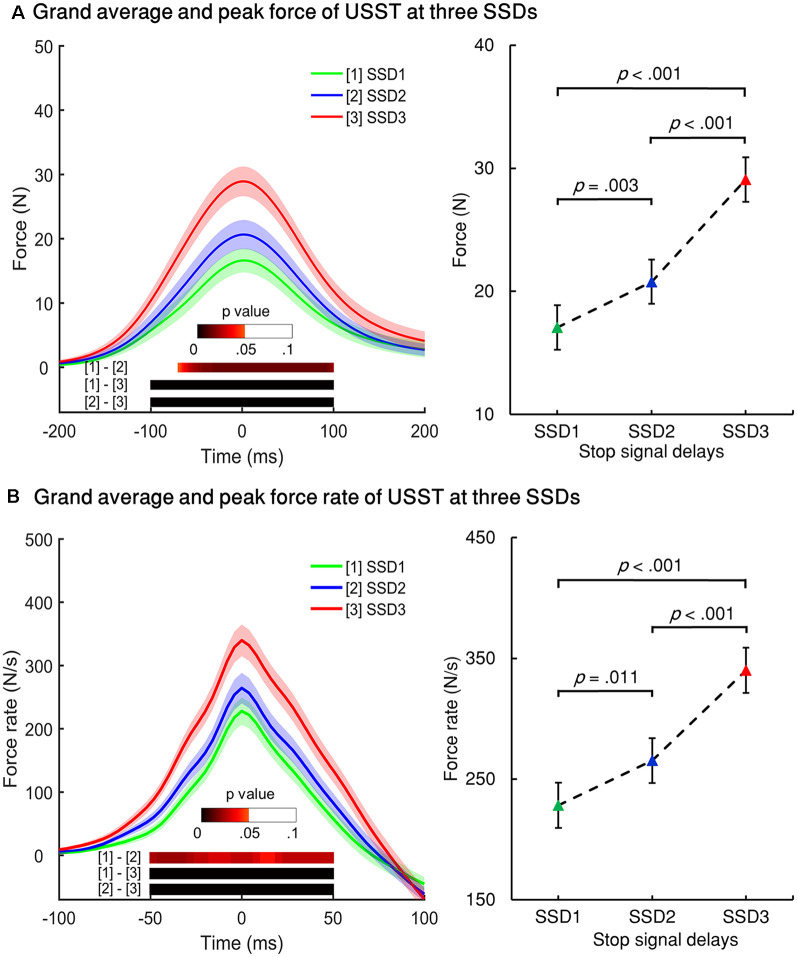
Force and force rate values for the three SSDs for non-canceled response trials. **(A)** Left panel: grand averages of all participants’ force from non-canceled responses were aligned to the peak force for three SSDs. Right panel: peak force of the three SSDs. **(B)** Left panel: grand averages of force rate were aligned to the peak force rate of USST for the three SSDs. Right panel: peak force for the three SSDs. The color bars indicate the level of *p*-value derived from the results of the *post-hoc* pairwise point-to-point comparisons (paired *t*-test for two conditions for each time point, FDR corrected) across three conditions. Shaded areas along with the curves represent 95% confidence intervals of peak force or peak force rate across conditions.

The force/force rate value of USST across the three SSDs were aligned to their corresponding peak force/force rate and the analysis was subjected to repeated measures ANOVA in a point-by-point comparison (left panel of Figures 5A,B). The gray bottom bar shows the significance (FDR corrected) of paired *t*-tests for the two conditions at each time point within the selected time windows ranging from −100 to 100 ms of the onset time of peak force and −50 to 50 ms of the peak force rate. The results indicate that the force and force rate increased with increasing SSDs for the non-canceled responses. To account for novel measurement indices of motor inhibitory control, the peak force and peak force rate of each SSD were further analyzed. Significant main effects for the peak force, *F*_(1.394,26.487)_ = 45.348, *p* < 0.001 (partial *η*^2^ = 0.705) and peak force rate, *F*_(1.256,23.871)_ = 38.049, *p* < 0.001 (partial *η*^2^ = 0.667) for non-canceled responses were observed (right panel of Figures 5A,B). Both the peak force and force rate for SSD3 were significantly larger than that of SSD1 and SSD2 (all *p* < 0.001). Also, the peak force for SSD2 was significantly larger than that of SSD1 (*p* = 0.003); so was the peak force rate (*p* = 0.011). These results show that the peak force and peak force rate increased with step-up of SSDs for non-canceled responses and thereby suggests that these measures and their dynamic characteristics can be used for gauging the inhibition function in addition to the conventional non-canceled rate.

#### Modulation of Force Rate and Reaction Time in Cont_Go Trials

**Figure 6:**
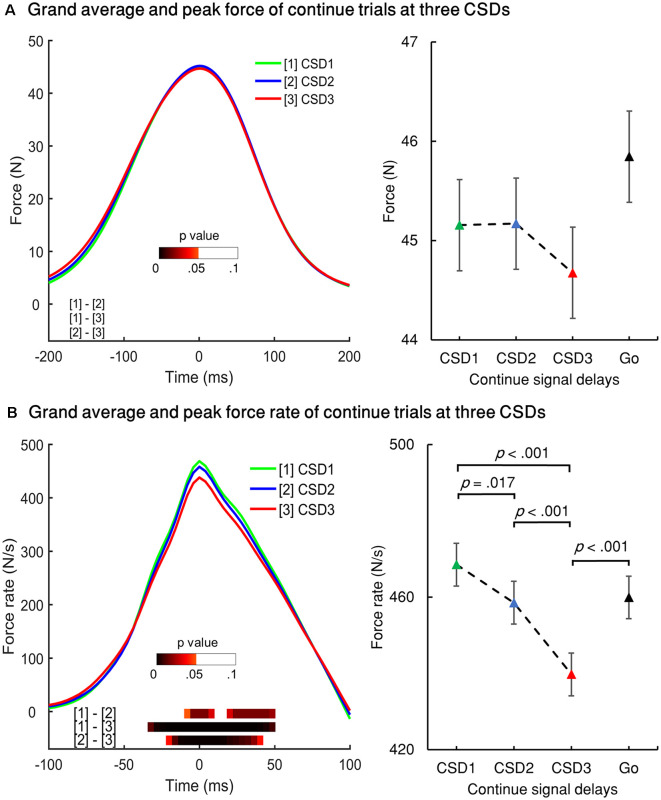
Force and force rate values for the three continue signal delays (CSDs) for Cont_Go trials. **(A)** Left panel: grand averages of all participants’ force aligned to peak force for the three CSDs for Cont_Go trials. Right panel: peak force of the three CSDs and go condition. **(B)** Left panel: grand averages of force rate aligned to the peak force rate of Cont_Go trials for the three CSDs. Right panel: peak force for the three CSDs and go condition. The color bar shows the level of differences between pairs of conditions (paired *t*-test for two conditions for each time point, FDR corrected); Error bars represent 95% confidence intervals.

RT, force, and force rate for Cont_Go trials were analyzed across CSDs using repeated-measures ANOVA. This showed a main effect for mean RT, *F*_(1.555,29.538)_ = 9.883, *p* = 0.001 (partial *η*^2^ = 0.342). *Post-hoc* tests with Bonferroni correction showed that the RT in CSD3 (*M* = 392.7, SEM = 13.2) was significantly faster than that of CSD1 (*M* = 419.9, SEM = 9.8), *p* = 0.008 and CSD2 (*M* = 412.7, SEM = 11.8), *p* = 0.004 but no significant difference was seen for CSD1 compared to CSD2 (*p* = 0.6). Interestingly, the go RT was significantly faster than that of CSD1 (*p* < 0.001), CSD2 (*p* < 0.001), and CSD3 (*p* = 0.006). There was no significant difference in force values across the three CSDs (left panel of Figure 6A). The peak force go was also no significant different when compared for the three CSDs. In the left panel of Figure 6B, the color bar in the bottom of the curves accounts for the significance (FDR corrected) of paired *t*-tests for the two conditions for each time point within the selected-time windows. The mean peak force rate significantly differed across CSDs, *F*_(2,38)_ = 30.404, *p* < 0.001 (partial *η*^2^ = 0.615). The peak force rate for CSD1 (*M* = 468.5, SEM = 22.1) was significantly larger than that of CSD2 (*M* = 458.5, SEM = 22.2), *p* = 0.017 and CSD3 (*M* = 439.7, SEM = 21.7), *p* < 0.001. The peak force rate for CSD2 was also significantly larger than that of CSD3 (*p* < 0.001). Comparing the go condition (right Figure 6B), the go peak force rate was larger than that of CSD3 (*p* < 0.001). However, there was no significant difference in force rate between go and CSD1; and between go and CSD2.

### Event Related Mode (ERM)

#### Stop vs. Cont_Go

**Figure 7:**
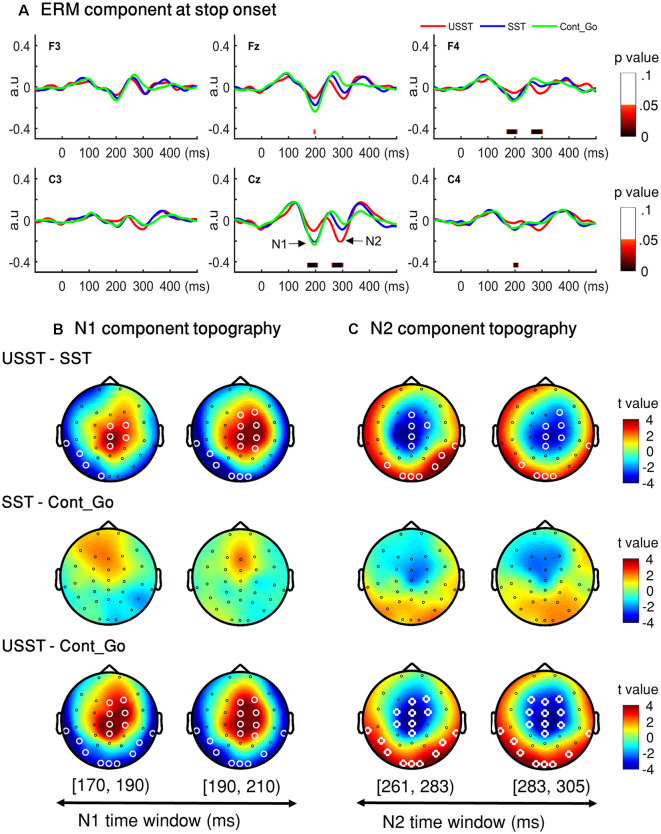
N1 and N2 results. **(A)** The grand average waveform of successful stop trials (SST) and unsuccessful stop trials (USST) and Cont_Go trials (Cont_Go) in the frontal-central electrodes (F3, Fz, F4, C3, Cz, and C4). The zero point on the *x*-axis represents the stop-signal onset. The *y-axis* is the normalized amplitude (a.u.). The EEG data of all trials were normalized by dividing by their standard deviation in each channel, resulting in a unit-free measure of amplitude. The color bar in the bottom of the plots shows the level of differences (FDR corrected) derived from the one-way ANOVA for the three conditions at each time point within the time window. Panels **(B,C)** show the topography indicating the differences between pairwise comparison across conditions in the time window of N1 (170–209 ms) and that of N2 (261–304 ms), respectively. The latency time window was divided into two time bins and each topography represents one time-bin. The right color bar shows the *t-value* (red color represents positive *t*-value and blue color represents negative *t*-value). EEG channels highlighted by a white circle indicate a significant difference between conditions in that channel, *p* < 0.05, two-tailed CBnPP test.

To choose the time window of each component (N1 and N2) for further analysis, one-way ANOVA with three conditions (SST, USST, and Cont_Go trials) was performed on the ERM amplitudes within the time window 0–500 ms after stop/Cont_Go signal onset for each data point in the frontal-central channels (F3, Fz, F4, C3, Cz, and C4). The color bar shows the level of main effects (FDR corrected) derived from one-way ANOVA of the three conditions at each time point within the time window (Figure 7A). Two of the largest time windows, the N1-related (170–209 ms) and N2-related components (261–304 ms) across six channels were selected. To calculate the topographic ERM differences among conditions, the latency time range was divided into two time-bins and the mean amplitude of N1 and N2 were calculated by averaging all the time points within the selected time-windows for each bin, each condition, each channel, and each participant.

Topographic results of the N1 amplitude component, USST condition showed a significant increment in the mid-frontal site and a significant decrement at occipital and temporal sites compared to the SST and Cont_Go conditions, *p* < 0.05, *N* = 20, two-tailed CBnPP test (top and bottom row of Figure 7B). In other words, the USST elicited relatively more positive potential than both SST and Cont_Go trials at mid-frontal. At occipital and temporal sites, the USST trials elicited relatively more negative potential than both SST and Cont_Go trials, *p* < 0.05, *N* = 20, two-tailed CBnPP test.

The N2 component displayed an effect similar to the N1 component but in the opposite direction, i.e., the N2 is reduced at mid-frontal and enhanced at occipital and temporal for USST compared to both SST and Cont_Go, *p* < 0.05, *N* = 20, two-tailed CBnPP test (top and bottom row of Figure 7C). Specifically, the USST trials elicited relatively more negative potential than both SST and Cont_Go trials at mid-frontal sites. At the occipital and temporal regions, the USST trials elicited a more positive potential than SST and Cont_Go trials. No significant difference was observed between SST and Cont_Go trials for both N1 and N2 time windows (middle rows of Figures 7B,C).

#### ERM Results of N1 and N2 in Partial and Full USST

**Figure 8:**
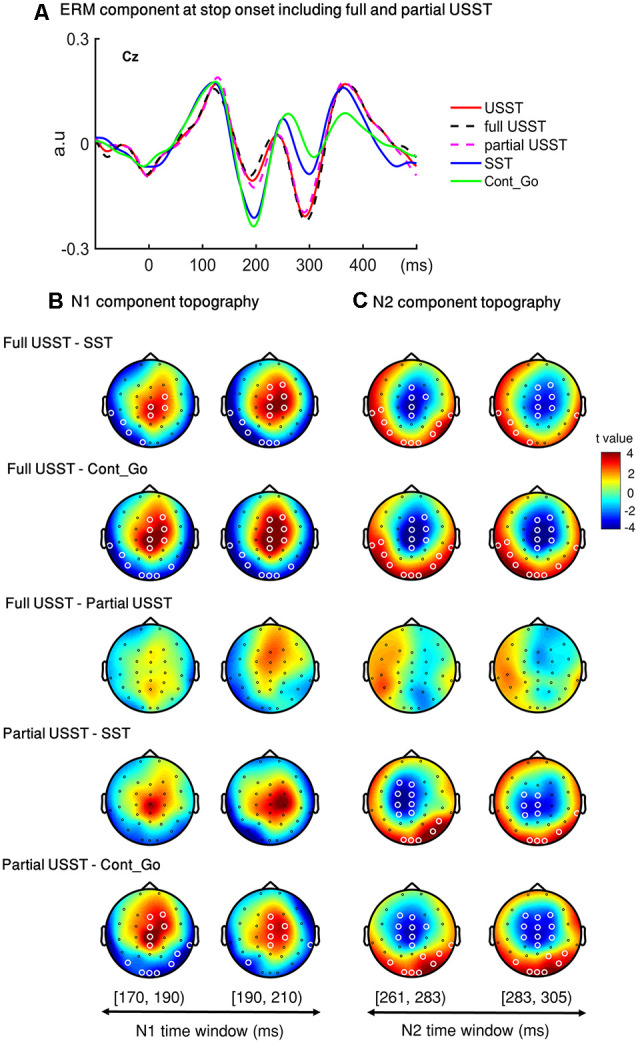
Event-related mode (ERM) results of N1 and N2 in partial and full USST. **(A)** The grand average waveform of successful stop trials (SST), full unsuccessful stop trials (full USST), partial unsuccessful stop trials (partial USST), Cont_Go trials (Cont_Go), and unsuccessful stop trials (USST; combined full and partial USST) at Cz. The zero point on the *x*-axis represents the stop-signal onset. The *y-axis* is the normalized amplitude (a.u.). Panels **(B,C)** show the topography indicating the differences between pairwise comparison across conditions in the N1 (170–209 ms) and N2 (261–304 ms) time windows respectively. The latency time window was divided into two time bins with each topography representing one time-bin. The right color bar shows the *t-value* (red color represents positive *t*-value and blue color represents negative *t*-value). EEG channels highlighted by a white circle indicate the significance between conditions in that channel, *p* < 0.05, two-tailed CBnPP test.

To look into details of the electrophysiological correlates of the inhibitory process, the N1 and N2 were analyzed separately in the partial USST and full USST trials. The time window of N1 (170–209 ms) and N2 (261–304 ms) were selected. The latency time window was divided into two time-bins to contrast the conditions. The grand average waveform of the partial and full USST in Cz is shown in Figure 8A. The amplitude of the full USST condition was significantly smaller relative to SST/Cont_Go in N1, *p* < 0.05, *N* = 20, two-tailed CBnPP test. In contrast, the full USST condition was significantly larger compared to SST/Cont_Go in N2, *p* < 0.05, *N* = 20, two-tailed CBnPP test. The full USST condition also revealed a significantly smaller amplitude than partial USST in N1, *p* < 0.05, *N* = 19, two-tailed CBnPP test. However, the full USST and partial USST trials displayed no significant difference for the N2 component. Moreover, no significant difference was observed between partial USST and SST trials and also between partial USST and Cont_Go trials in both N1 and N2 time windows at Cz.

In the topography of the amplitude of N1 component, the full USST condition revealed a significant increment in the mid-frontal site and a significant decrement at occipital and temporal sites relative to the SST and Cont_Go conditions, *p* < 0.05, *N* = 20, two-tailed CBnPP test (the top and the second row of Figure 8B). The full USST condition also showed a significant increment in the mid-frontal site compared to the partial USST condition, *p* < 0.05, *N* = 19, two-tailed CBnPP test (the third row of Figure 8B).

The N2 component displayed an effect similar to the N1 component but in an opposite direction, i.e., the N2 was reduced at mid-frontal and enhanced at occipital and temporal locations for full USST compared to both SST and Cont_Go, *p* < 0.05, *N* = 20, two-tailed CBnPP test (see the top and the second row of Figure 8C). No significant difference was observed between the full and partial USST trials in N2 time windows (the third rows of Figure 8C). No significant difference was observed between partial USST and SST (the fourth row of Figures 8B,C); and between partial USST and Cont_Go trials (bottom row of Figures 8B,C) in both N1 and N2 time windows. The comparison of each of the paired conditions in N1 and N2 is summarized in Table 2.

**Table 2:** The comparisons between each paired conditions in N1, N2, and LRP.

	Full USST	Partial USST	SST	Cont_Go	USST	Go
The comparisons of each paired conditions in the N1 component						
Full USST	–	*	*	*	–	–
Partial USST	*	–	n.s	n.s	–	–
SST	*	n.s	–	n.s	*	–
Cont_Go	*	n.s	n.s	–	*	–
USST	–	–	*	*	–	–
The comparisons of each paired conditions in the N2 component
Full USST	–	n.s	*	*	–	–
Partial USST	n.s	–	n.s	n.s	–	–
SST	*	n.s	–	n.s	*	–
Cont_Go	*	n.s	n.s	–	*	–
USST	–	–	*	*	–	–
The comparisons of each paired conditions in LRP
Full USST	–	*	*	n.s	–	n.s
Partial USST	*	–	*	*	–	*
SST	*	*	–	*	*	*
Cont_Go	n.s	*	*	–	*	n.s
USST	–	–	*	*	–	*
Go	n.s	*	*	n.s	*	–

Note: USST: combined full and partial USST; n.s, no significant difference; “–”, not applicable; *p < 0.05.

### Error-Related Negativity (ERN) Results

**Figure 9:**
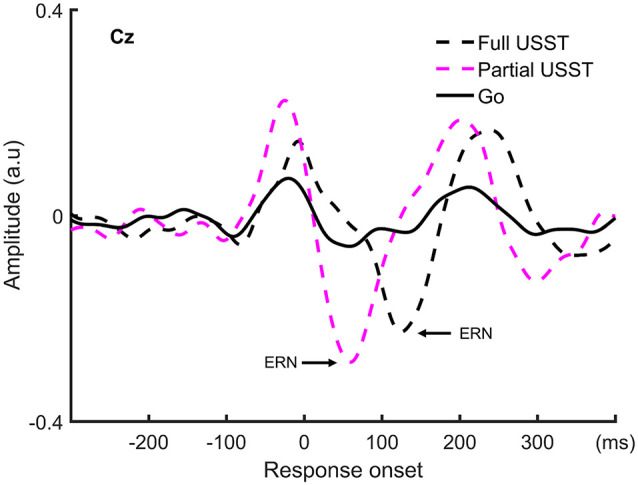
The grand average waveform at Cz for partial USST, full USST, and go condition. The zero point on the *x*-axis represents the response onset.

To understand the late temporal processing involved in inhibitory control, the ERN analysis was categorized into partial USST and full USST trials. The ERN grand average waveform at Cz for partial USST and full USST is shown in Figure 9. The latency of the positive peak occurred at −25 ms for partial USST and −7 ms for full USST. The amplitude of the positive peak was calculated by the mean amplitude from −45 to −5 ms for partial USST and from −27 to 12 ms for full USST. The amplitude of the ERN was significantly larger in partial USST (*M* = −0.55 a.u, SEM = 0.06) compared with full USST (*M* = −0.39 a.u, SEM = 0.04), *t*_(18)_ = −2.743, *p* = 0.013. Moreover, the ERN amplitude of partial and full USST was larger than go (*p* < 0.001). The latency of the peak ERN for partial USST (*M* = 74.4 ms, SEM = 8.2) was significantly earlier relative to the full USST (*M* = 130.0 ms, SEM = 5.6), *t*_(18)_ = −7.423, *p* < 0.001.

### Source Localization of ERM

**Figure 10:**
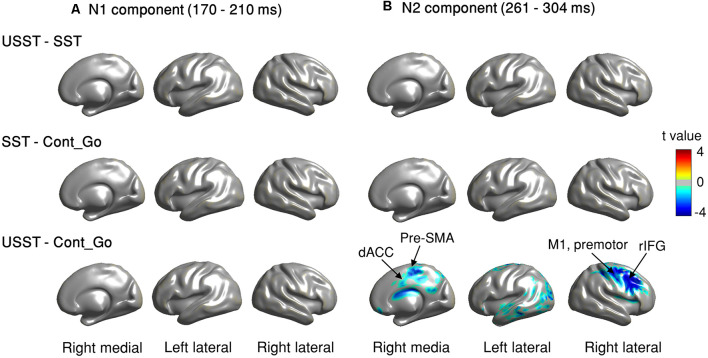
**(A,B)** Source level ERM contrast of comparisons in N1 and N2 time windows. Brain regions displayed with colors (except gray, color shades denote *t*-values) indicate that the difference of N1 and N2 in these regions was significant, *p* < 0.05, *N* = 20, CBnPP test.

To identify the brain regions associated with inhibition and/or attention capture, we estimated ERM and employed CBnPP tests between each pair of conditions at the source level. We focused on the N1-related (170–209 ms) and N2-related components (261–304 ms) following the sensor level time window. In Figure 10, the contrast was observed at the right lateral, left lateral, and medial views to illustrate the brain areas in which the source-level ERM was significant for each paired comparison in N1 and N2.

For the N1 component, no significant difference was observed between USST and SST (top row of Figure 10A); and between SST and Cont_Go trials (middle row of Figure 10A). Also, the USST and Cont_Go displayed no significant difference in the N1 component (bottom of Figure 10A). In contrast, the USST condition displayed a significantly decreasing activation relative to the Cont_Go for N2 component, *p* < 0.05, *N* = 20, two-tailed CBnPP test (bottom row of Figure 10B). The brain areas representative of the significance included right M1, rIFG, pre-motor cortex, and dACC in addition to some other temporal and occipital areas. No significant difference was recorded between USST and SST trials (top row of Figure 10B); and between SST and Cont_Go (middle row of Figure 10B).

### LRP Results

**Figure 11:**
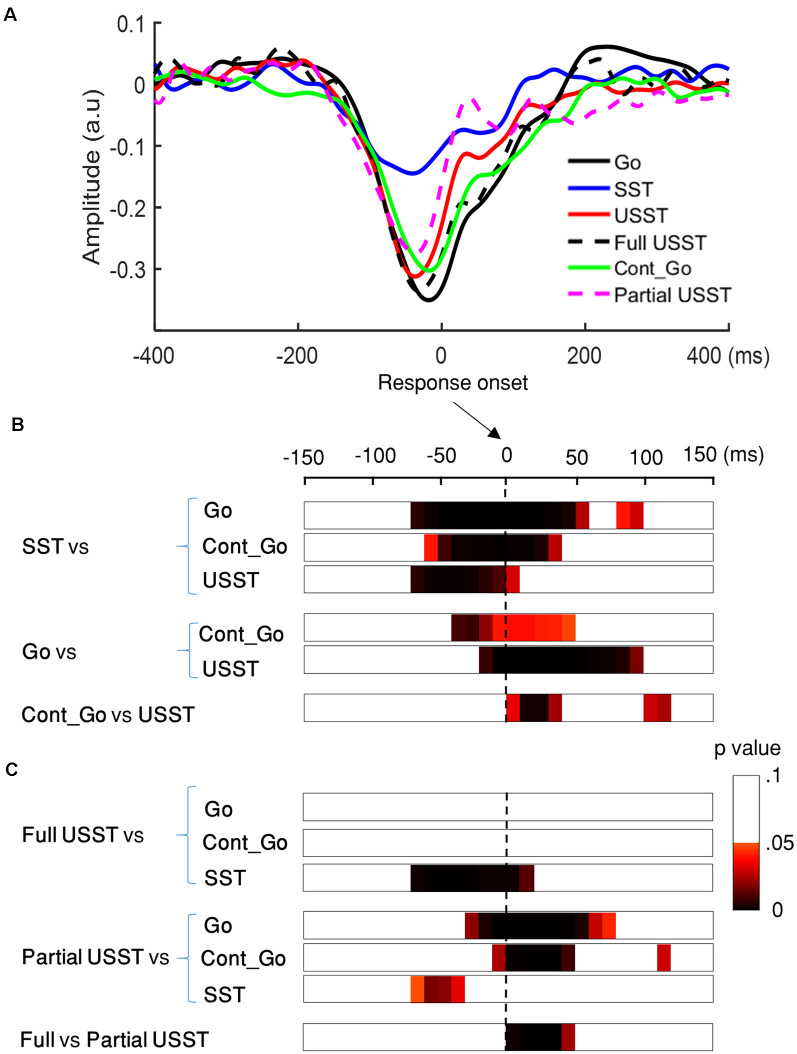
Lateralized readiness potential (LRP) results of go, SST, Cont_Go, and USST condition. **(A)** LRP time-locked to the response onset for the C3/C4 electrodes for each condition in which go, SST, Cont_Go, full USST, partial USST, and USST (combined full and partial USST). The *y*-axis is the normalized amplitude (a.u.). LRPs were obtained for each trial (go, Cont_Go, USST, full USST, and partial USST) relative to response onset. For SST trials, the LRP waveform was aligned to (SSRT + SSD2). **(B)** The color bar indicates the level of difference between pairs of conditions (two-tailed CBnPP test). Combined full and partial USST were used in the case of the USST condition. The dashed vertical line indicates response onset. **(C)** The color bar indicates the level of differences between pairs of conditions (two-tailed CBnPP test), USST was divided into full and partial USST for further comparisons.

To confirm the LRP existence across all conditions (USST, Cont_Go, go, SST), two-tailed cluster-based permutation tests were conducted for LRP amplitude in the 300 ms interval (150 ms before and after the onset). The concurrent time-window (−80 to 10 ms; not shown in Figure 11) differed significantly from 0 (the baseline) for all conditions, *p* < 0.01, *N* = 20, two-tailed CBnPP test. This indicates the presence of LRP across all conditions i.e., including the successful stop condition.

The LRP amplitudes were smaller for SST trials relative to go, Cont_Go and USST trials, *p* < 0.05, *N* = 20, two-tailed CBnPP test (first to third rows of Figure 11B). LRP amplitudes were larger for the go trials than for the Cont_Go and USST trials, *p* < 0.05, *N* = 20, two-tailed CBnPP test (the fourth and fifth row of Figure 11B). LRP amplitudes were significantly different between USST and Cont_Go trials, *p* < 0.05, *N* = 20, two-tailed CBnPP test (bottom row of Figure 11B). We also found that LRP amplitude was larger for the go trials than for the Cont_Go at the go stimulus, *p* < 0.05, *N* = 20, two-tailed CBnPP test (see Supplementary Material).

When the USST was further sub-divided into full and partial USST, the mean LRP of full USST was not significantly different from the mean LRPs of go and Cont_Go trials (the first and the second row of Figure 11C). LRP amplitudes were significantly different between full USST and SST trials, *p* < 0.05, *N* = 20, two-tailed CBnPP test (the third row of Figure 11C). The LRP amplitude was smaller for partial USST than for go, and Cont_Go trials, *p* < 0.05, *N* = 19, two-tailed CBnPP test (the fourth and the fifth row of Figure 11C). The LRP amplitude was larger for partial USST than SST trials, *p* < 0.05, *N* = 19, two-tailed CBnPP test (the sixth row of Figure 11C). However, the LRP amplitude was smaller for partial USST than full USST trials, *p* < 0.05, *N* = 19, two-tailed CBnPP test (the bottom row of Figure 11C).

**Figure 12:**
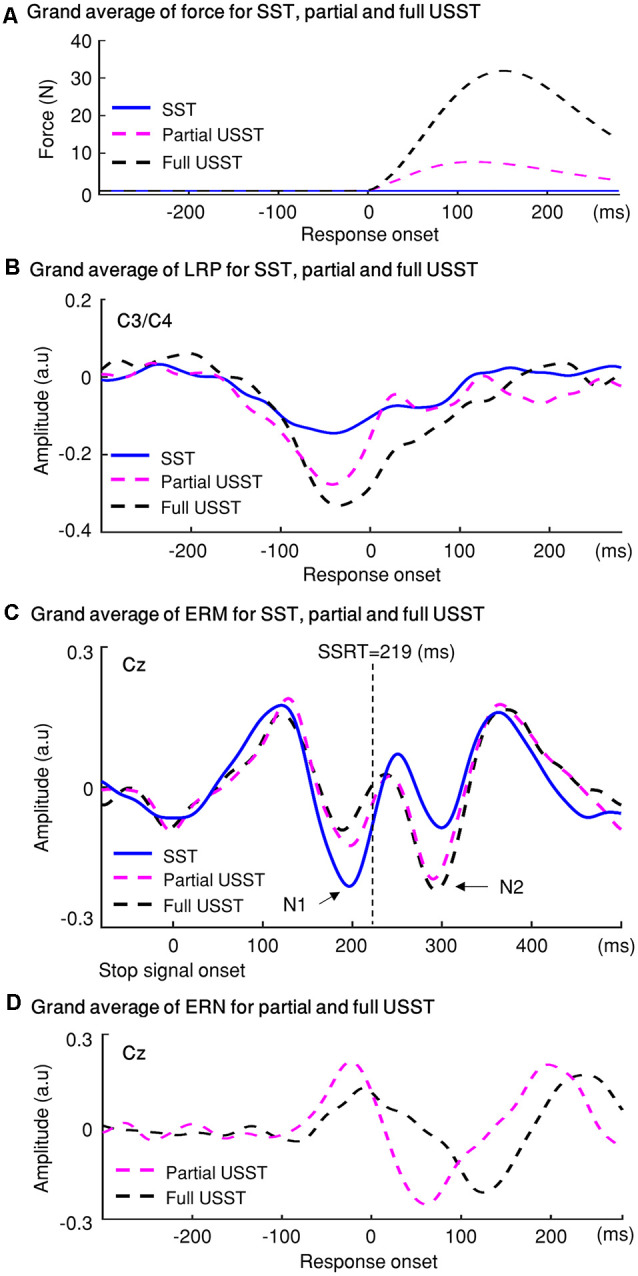
The temporal evolution of the stopping process and their electrophysiological signatures: LRP, ERM, and error-related negativity (ERN). **(A)** Grip force: the grand averages of forces aligned to response onset for the three conditions: SST, partial USST, and full USST. **(B)** LRP: the grand average LRP waveform of SST, partial USST, and full USST in C3/C4. **(C)** ERM: the grand average ERM waveform of SST, partial USST, and full USST in Cz. **(D)** ERN: the grand average waveform at Cz for ERN in partial USST and full USST.

The findings for stop trials are summarized in Figure 12. The force value of full and partial USST was significantly greater than those of SST. Also, the force value of full USST was significantly larger than those of partial USST (Figure 12A). The same pattern was found in the LRP amplitude (Figure 12B). The LRP amplitude was larger for full and partial USST than SST trials. LRP amplitude of full USST was significantly larger than those of partial USST after the response onset. Regarding ERM (Figure 12C), the full USST condition was significantly smaller in comparison to partial USST and SST in N1. In contrast, the full USST condition was significantly larger compared to SST in N2. No significant difference was observed between the SST and partial USST trials in both N1 and N2 time windows in Cz. The amplitudes of the ERN were larger in partial USST compared with full USST. The latency of the peak ERN for partial USST was significantly earlier than for full USST.

## Discussion

In this study, we utilized grip force to aid in developing new measures of motor inhibitory control, with it providing a gradient and finer estimate of inhibitory control processes. Additionally, a modified version of the stop-signal task was used to control for attentional capture. A major limitation of using the conventional stop-signal task to investigate motor inhibition mechanisms is the stop-signal may evoke both attentional capture and response inhibition processes concurrently (Sharp et al., 2010; Lee et al., 2016). This issue can be resolved by employing a modified version of the stop-signal task by introducing “continue go” trials (Cont_Go) to control any effects caused by attentional capture. Furthermore, the common-used temporal time marks for gauging electrophysiological correlates may also not be precise in the conventional stop-signal task because the go trials may utilize a rather arbitrary categorization of “fast go trials”/“slow go trials” to compare with successful and unsuccessful stop trials for the ERP components (Kok et al., 2004; Lo et al., 2013). By adding Cont_Go trials to the task, direct time stamps for precise comparison of ERP components across stop, Cont_Go and go trials may be obtained.

### New Behavioral Measures of Motor Inhibitory Control

Our results have demonstrated that the peak force and peak force rate of unsuccessful stop-signal trials were increased with increments of SSDs. These functional dynamics between SSDs and force measures can represent new indices for gauging the degree of urgency in motor inhibitory control. Furthermore, the reaction time and force rate of Cont_Go trials decreased with increased CSDs. This indicates that the motor response time and the force rate are functions of preparation to deal with a novel/infrequent stimulus in the environment.

The smaller peak force/peak force rate observed in USST than that in go trials is in line with reports from previous studies (de Jong et al., 1990; Ko et al., 2012). This observation provides evidence that the presentation of a stop-signal interrupts the primary task response in a disruptive manner. It can be inferred that although stop processes under USST lost the race to the go response processes, lower force and force rate show the residual effects of the inhibition process. Carbonnell and colleagues suggested that participants may be inhibiting errors as they are being executed (Carbonnell and Falkenstein, 2006). McGarry and Franks (1997) also suggested that response inhibition should be viewed in terms of a disruptive process in which an EMG activity burst was quite short-lived relative to the full response trials. In the current study, we intended to measure the graded nature of motor inhibition. Following our hypothesis, the peak force/force rate of USST increased as a function of SSD and the peak force/force rate of partial USST also increased as a function of SSD. In stop trials, the longer the SSD, the more difficult it is to inhibit a response. We can assume that in the case of shorter SSDs, the error correction processes occur enough early to block the motor execution process even though the motor response has already started, while with longer SSDs it is either too late to effectively correct the response or too late for the correction to occur and block the motor execution process. Hence, the force/force rate increases as a function of SSD, and peak force and peak force rate can offer new estimates of motor inhibitory control.

### Modulation of Reaction Time and Force Rate for the Novel/Infrequent Stimuli

To comply with the assumptions of the horse race model, we defined the full USST by objectively and systematically choosing USST trials in which the RTs were faster than those in the go trials (Wang et al., 2013; Bissett and Logan, 2014). We found that the RTs of partial USST trials were slower than RTs in both go and full USST (de Jong et al., 1990). The results showed that full USSTs were responses made too fast for them to be withdrawn, with those trials positioned in the left part of the go RT distribution. The middle part of the go RT distribution was more in line with partial USST. In the task, the participants also took a longer time to respond to the Cont_Go trials compared to go trials, showing that the processing of these two trials was different. Sharp et al. (2010) proposed that Cont_Go signals might trigger incomplete inhibitory processing and decay go activation that might lead to a delayed response, while go trials might trigger an imperative motor response. In another study, Cai and Leung (2011) employed modified stop-signal tasks (a stop-signal task and a not-stop task) to separate the stop-related processes from the attentional capture of an infrequent stimulus from process related to rule retrieval. The longer RT of not-stop trials (similar to the Cont_Go trials here) compared to the RT for go trials was observed. Moreover, this revealed differential involvement of the right dorsal IFG and the left anterior IFG in attentional capture and rule retrieval, respectively, suggesting that an infrequent stimulus (Cont_Go signal) may also involve rule retrieval. We also observed that the reaction time and force rate of Cont_Go trials decreased with increments of CSDs. It can be inferred that under shorter CSDs, the extent of preparation for go response may have been less than that under longer CSDs before the Cont_Go signal appear. The shorter CSDs might lead to a transient stop and re-initiation of the go response. For the longer CSD condition, the signal appears late and participants are more prepared to make a go response (Wang et al., 2013). Therefore, for the longer CSDs, the response was executed faster than for the shorter CSDs, resulting in a faster Cont_Go RT. The variations observed in the RT and force rate on Cont_Go trials that were functions of delay in presentation of a novel/infrequent stimuli in the environment suggests that the go activation might have a non-linear function with respect to time.

### The Electrophysiological Characteristics of Stop and Continue Go

In the electrophysiological data for the stop and continue go conditions, we found that there was a smaller N1 amplitude in USST compared to the SST as well as to the Cont_Go condition, while no significant difference was observed between the SST and the Cont_Go condition. This suggests that the N1 component can be categorized as an index for measuring the early processes of inhibitory control. The N1 wave was typically indexed for a discrimination process—the process of differentiation between two or more types of stimuli, but may not be associated with motor responses (Vogel and Luck, 2000). We found the appearance of the N1 component in response to both Cont_Go and SST, with no differences in N1 amplitude for the two conditions. This suggests that the N1 component may also appear as a common discrimination process associated with Cont_Go and stop-signals, so might occur in response to novel signals in the environment. Moreover, the participants took a longer time to respond to the Cont_Go trials compared to go trials indicating that incomplete inhibitory processing is triggered by Cont_Go signals (Sharp et al., 2010; Wessel and Aron, 2017) and occurs in the early processing and discrimination of the signal (van de Laar et al., 2010). These findings suggest that the N1 component of continue go trials may relate not only to the discrimination processes but also to the motor inhibitory processes. Ramautar and colleagues showed that there was no difference in N1 between SST and USST conditions and therefore proposed that the N1 component might be merely associated with the sensory processing of the stop-signal, but not with motor processing (Ramautar et al., 2006). If that was the case, there should not be any difference in the N1 component across three types of conditions (i.e., SST, USST, and Cont_Go). In contrast, in the current study, we observed significant differences in N1 between USST and SST as well as between USST and Cont_Go trials suggesting that the N1 component is an endogenous (goal-driven) component. The N1 wave of USST was smaller than SST and Cont_Go trials in mid-frontal regions. In a previous study, enhancement of the N1 component for SST compared with USST suggested that inhibitory performance was affected by the strength of sensory processing of the stop stimulus (Bekker et al., 2005). Kirmizi-Alsan et al. (2006) by utilizing a go/no-go task found a significant N1 amplitude increase in no-go trials compared with go trials, with the component being associated with inhibition. This pattern of results suggests that the N1 component may relate not only to the discrimination processes but also to the motor inhibitory processes, due to the overlapping and successful inhibitory processes in SST and Cont_Go conditions.

The results relating to the N2 component from the ERM analysis were similar to the N1 component but were opposite in the direction of effects. The N2 was larger during USST in comparison to Cont_Go trials and SST, but not different for SST and Cont_Go trials. This pattern is consistent with some conventional findings in which enhancement of N2 was found in failed trials in comparison to successful ones, thereby indicating its role in inhibitory processes (Kok et al., 2004; Ramautar et al., 2004, 2006). Similar findings are reported in response conflict between concurrently activated responses (e.g., withhold vs. execute; (van Boxtel et al., 2001; Botvinick et al., 2004; Huster et al., 2013). In the present study, the role of N2 in inhibition or conflict can be delineated by comparing the SST and Cont_Go conditions because both the stop-signal and the Cont_Go signal carry similar attentional and sensory processing demands, but only the stop-signal induces neural processes involved in canceling the planned go response.

Therefore, if the N2 component is associated with response conflict between go and stop, there would be a difference in N2 amplitude between SST and Cont_Go trials. In contrast to this prediction, no difference between Cont_Go and SST was observed. Also, the N2 component in USST trials was significantly larger than in SST and Cont-Go trials. These results suggest that the N2 component is associated with the inhibition process rather than with response conflict (Figure 7).

In terms of the sources of the ERM components, the source localization data showed rIFG, pre-motor cortex, right M1, dACC, and pre-SMA were associated with greater activation of the N2 component for Cont_Go in comparison to USST trials. The patterns of the results are in line with previous functional neuroimaging studies (Rubia et al., 2003; Aron et al., 2004). On the other hand, no significant difference was observed between SST and USST as well as between SST and Cont_Go trials in rIFG, right anterior insular (rAI), and pre-SMA. Thus, we did not find evidence supporting the specific role of these areas in the inhibition process (Sharp et al., 2010; Swick et al., 2011; Cai et al., 2014). Sharp et al. (2010) showed SST was associated with activation of the rIFG and rAI as well as the pre-SMA. However, the activity of pre-SMA, not rIFG and rAI, was greater on SST trials than on Cont_Go trials. These findings suggest that the rIFG supports attentional capture and pre-SMA suppresses the ongoing action. Moreover, meta-analyses (Swick et al., 2011; Cai et al., 2014) showed that the highest activation was observed in rAI as well as this activation being greater on USST trials than SST trials. The findings suggest that the rAI is particularly important for detecting salient events. This inconsistency may be due to the difference in experimental procedures or participant samples. As a result, comparing EEG and fMRI effects are still limited. For instance, Cai et al. (2014) reported the fMRI results from the conventional stop-signal task and there was significantly greater activation in the right anterior insular (rAI) for USST than SST. We used a modified version of the stop-signal task which had added continue go trials. However, the neural activities associated with the inhibition process may not strong enough to be reflected in the EEG signal compared to the fMRI signal (Cai et al., 2014). This is an important issue that needs to be further explored in the future, ideally by measuring EEG and fMRI simultaneously.

### Effect of Novel/Infrequent Stimuli on Central Processing

A smaller LRP amplitude was observed in Cont_Go trials than in go trials, indicating an influence of Cont_Go signals on central motor processing. The LRP is conventionally attributed to preparation for executing hand responses. In this task, one hand was used to respond in a trial. If Cont_Go signals affect LRP, the LRP in Cont_Go trials would be different from that of the go trials. A smaller amplitude LRP was seen in Cont_Go trials compared to go trials suggesting that when the go condition is used as a baseline, the motor system is affected by the Cont_Go condition by decreasing the amplitude of LRP. The smaller LRP in the Cont_Go condition can be associated with the re-initiation of the motor response after the Cont_Go signal appeared. Thereby the Cont_Go condition induces different processes than the go condition in central electrophysiological and behavior measures.

### Differential Characteristics of Full and Partial USST

The primary advantage of force-based measurement is its ability to capture the commonly occurring “partial” unsuccessful trials that are highly relevant in extending the current understanding of the behavioral and neural electrophysiological mechanisms of motor inhibition. The peak force of partial USST was smaller than that in full USST. The appearance of partial responses illustrates that the response activation was interrupted before proceeding to the full response. The LRP amplitude of full USST was larger than partial USST after response onset, and the ERN amplitude of partial USST was also larger than the full USST. Additionally, the ERN occurs earlier for the partial USST than full USST trials. These results suggest that the ERN and LRP components may reflect the “early” error correction processes. Furthermore, the functional significance of the N1 and N2 components of partial USST was also examined. A relatively smaller N1 amplitude was observed for full USST than for the partial USST, Cont_Go, and SST. A significantly larger N2 amplitude was recorded for the full USST trials compared to the SST and Cont_Go trials. However, no significant difference was found between partial USST and full USST in N2. Moreover, partial USST was not significantly different from the SST and Cont_Go trials for their N1 and N2 amplitudes. This suggests that the partial USST processes were more similar to those involved in SST and Cont_Go trials rather than that in full USST trials despite both partial and full USST trials being inhibition errors. This also implies that the N1 component of partial USST may potentially be associated with “early” inhibitory processes, whereas the N2 of partial USST may be an overlap between inhibition and error correction.

#### Patterns of Error Correction

The peak force of partial USST was smaller than that in full USST, which is in-line with previous studies (de Jong et al., 1990; Ko et al., 2012). The results from the current study tested the inhibition of partial USST trials at any time-point during its execution, thereby providing ample evidence contrary to the ballistic stage reported by De Jong and colleagues. Moreover, the partial USST may indicate that the error response activations could be inhibited before proceeding to full responses. In the aspects of electrophysiological signatures, there was a larger LRP amplitude for the go than the SST and partial USST trials, which is as per previous studies (de Jong et al., 1990). According to de Jong et al. (1990), the attenuated LRP reflects the effect of the central inhibitory mechanism on motor preparation. Therefore, the smaller LRP amplitude of partial USST trials indicated that the central mechanism contributed to the operation of inhibitory control. In the current study, we found that the LRP amplitude of full USST was larger than partial USST only after the response onset. It can be interpreted that, before their responses, the central inhibitory mechanism may have a similar effect on the motor preparation between full and partial USST. In contrast, after the initiation of response, the LRP amplitude of full USST was larger than partial USST. Previous studies suggested that the reduced activity of the motor system (inhibition) was associated with the reduced LRP amplitude (de Jong et al., 1990; van Boxtel et al., 2001). According to this line of logic, we can infer that the partial USST differs from full USST across central electrophysiological and peripheral behavior measures because the partial USST may involve a disruptive process that timely withdraws the so-called “ballistic” incorrect response.

A significantly larger LRP amplitude was observed in partial USST compared to SST from −70 to −30 ms. The different amplitudes indicated that partial USST may be comprised of an initial response then inhibition. This finding was strengthened by the subsequent comparison that revealed a smaller LRP amplitude for partial USST than go from −30 to 80 ms (Figure 11). These results illustrated a clear transient in partial USST from initial action to the rapid switch to inhibition of the action. These results also suggest that reduced motor activity in partial USST could reflect the error correction process.

The ERN amplitude of partial and full USST was larger than go, suggesting that ERN is associated with the error response (Gehring et al., 1993). We found that the ERN amplitude of partial USST was larger than that of full USST, which is consistent with the hypothesis of the response conflict model (Carter et al., 1998; Botvinick et al., 2001). It can, therefore, be assumed that the temporal overlap of the error response and correction responses is greater in partial USST than in full USST. Thus, a larger ERN amplitude was observed in partial USST compared to full USST. Furthermore, the ERN occurs earlier for partial USST than full USST, in line with a previous study (Carbonnell and Falkenstein, 2006). The result provides evidence that an error response would be a partial or a full response is associated with the timing of the ERN. It implies if the ERN occurs earlier, withhold of neural activations associated with an incorrect response before proceeding to full responses is more likely. Moreover, there was later ERN when errors were not corrected than when they were corrected. This could indicate that ERN must occur quickly if it is to assist in error correction (Fiehler et al., 2005; Hoffmann and Falkenstein, 2010). This combination of findings provides some support for the conceptual premise that the errors may be corrected for partial USST and uncorrected for full USST. Moreover, the ERN latency and amplitude could be modulated by the error correction processes.

#### The Roles of N1 and N2 on Partial Inhibitions

The appearance of partial responses illustrates that participants may inhibit error response activations before proceeding to full responses (Carbonnell and Falkenstein, 2006). Therefore the N1 component elicited by partial USST may relate to the inhibition process. We found that the N1 amplitude in partial USST was not significantly different from that of the SST and Cont_Go trials. In previous discussions, the N1 of SST and Cont_Go has been implicated in response inhibition. Thus, these findings suggest that similar inhibitory processes were deployed at the early stage in those types of trials. This finding was strengthened by the subsequent comparison that showed a smaller N1 amplitude for full USST than partial USST. Given that partial and full USST trials were elicited by stop-signals, the observed difference in N1 amplitude was not related to the stimulus signals. The results support the idea that the enhancement of N1 on partial USST reflects the inhibition of the response, which may be necessary for further processes such as error correction. In light of the N2 amplitude, no difference was found between SST and partial USST trials. This finding is contrary to some of the previous studies which illustrated a larger N2 on partial response trials than on successful trials (van Boxtel et al., 2001). This inconsistency may be due to the difference in tasks employed for the two experiments. For instance, Van Boxtel and colleagues combined the stop-signal and go/no-go task in their experiment. Another possible explanation for the inconsistency could be the difference in how partial responses were defined. In their study, the response was defined as partial if its force was smaller than the required level of force which is defined by 2–15% maximum of force [in comparison to (*M* − 3*SD) of go force defined in the current study]. We also observed no difference in the amplitude of the N2 on Cont_Go and partial USST. As discussed, the N2 of SST and Cont_Go reflected the inhibition process. Therefore, the N2 components of partial USST may be associated with inhibitory processes but not error detection. From the perspective of response inhibition, the N2 amplitude of partial USST could be smaller than in full USST. However, no difference in the N2 amplitude for full and partial USST was observed in the current study. The result raises the question about the subsequent error correction of the ongoing response in partial trials. The N2 of partial USST may overlap in its ERN, leading to an increase in the N2 amplitude of partial USST. These relationships can partly be explained by the continuation between N2 and ERN of partial USST trials. We observed the ERN component following the response onset of partial trials, illustrating that partial responses were associated with the error response. Moreover, the timing of the ERN coincided with the peak latency of N2. In its entirety, these results suggest that the N2 of partial USST may overlap inhibition and error correction.

### The Temporal Processes of Inhibitory Control

As shown in Figure 12, the dynamic stop process is initiated by the stop-signal and the stopping process transmits an inhibitory signal to the motor cortex with the electrophysiological components reflecting the temporal evolution of the processes. Firstly, the attenuated LRP indicates the effect of the central inhibitory mechanism on motor preparation. Moreover, both N1 and N2 components of SST reflects an index of motor inhibition. This advocate for the case of successful inhibition, accompanied by no force response. If the inhibitory signal appears too late, the central response processes are completely developed. Therefore, the response would be executed as a full response. For the later occurring inhibitory signal, the response activation was initiated. However, response activation was inhibited and corrected before it could proceed to full response. This situation represents the case for partial USST, characterized by a partial response. The LRP amplitude of partial USST was smaller than that of full USST while a larger ERN amplitude was observed in partial USST trials compared to full USST. Moreover, the ERN occurs earlier in the partial USST than in the full USST trials. These results illustrate that LRP and ERN components of the partial USST trial may be associated with fast error correction. For the N1 and N2 components, the N1 components of partial USST may potentially be associated with the inhibitory processes, whereas the N2 of partial USST may overlap across inhibition and error correction.

In conclusion, by using a pinch grip to measure the gradients of dynamic motor inhibition, we have developed alternative and precise indices of motor inhibition by introducing the force and force rate of the subject responses. Motor response (time and force) was a function of delay in the presentation of the Cont_Go signal. Furthermore, the Cont_Go signals effects were not only reflected in central processing (LRP) but also in the behavioral outcomes. The current electrophysiological findings also demonstrate that the early N1 component complements the existing N2 component as an early motor inhibition index. Moreover, the source estimation of N2 was associated with inhibition-related areas, such as right IFG, M1 and pre-motor cortex, and dACC. These findings suggest that the LRP and ERNs of partial responses are associated with error correction processes, whereas the N2 of partial response may be associated with an overlap of inhibition and error correction.

## Data Availability Statement

The raw data supporting the conclusions of this article will be made available by the authors, without undue reservation.

## Ethics Statement

The studies involving human participants were reviewed and approved by the Institutional Review Board of the Chang Gung Memorial Hospital, Linkou, Taiwan. The patients/participants provided their written informed consent to participate in this study.

## Author Contributions

TN, C-HJ, and W-KL designed the research. TN, C-HJ, W-KL, and NM analyzed the data. TN, C-YH, and SJ performed the experiments. All authors contributed to the article and approved the submitted version.

## Conflict of Interest

The authors declare that the research was conducted in the absence of any commercial or financial relationships that could be construed as a potential conflict of interest.
