# Supplementary material for: To Go or Not to Go: Degrees of Dynamic Inhibitory Control Revealed by the Function of Grip Force and Early Electrophysiological Indices
Source: Front Hum Neurosci. 2021 Jan 28;15:614978. doi: 10.3389/fnhum.2021.614978 (PMC7876446; doi:10.3389/fnhum.2021.614978)
Supplement: Supplementary file 1 [file Table_1.docx]

| 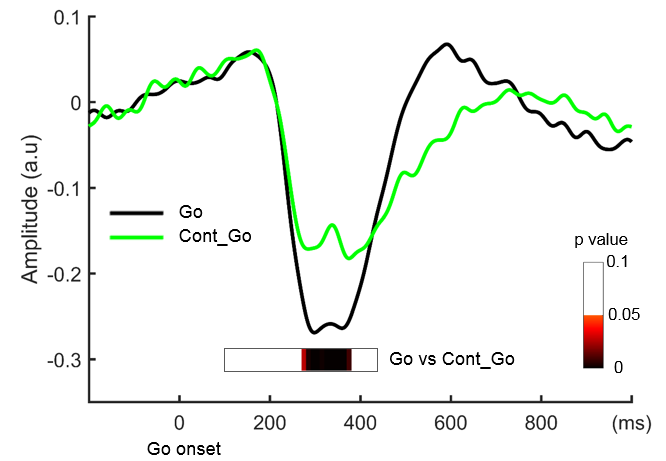 |
| --- |
| **Figure S1:** LRP time-locked to the go onset for the C3/C4 electrodes for go and Cont_Go conditions. The y-axis is normalized amplitude (a.u.). The color bar indicates the level of difference between go and Cont_Go of conditions (two-tailed CBnPP test). LRP amplitudes were larger for the go trials than for the Cont_Go trials, *p* < .05, *N* = 20, two-tailed CBnPP test |
